# Tetramerization Reinforces the Dimer Interface of MnSOD

**DOI:** 10.1371/journal.pone.0062446

**Published:** 2013-05-07

**Authors:** Yuewei Sheng, Armando Durazo, Mikhail Schumacher, Edith Butler Gralla, Duilio Cascio, Diane E. Cabelli, Joan Selverstone Valentine

**Affiliations:** 1 Department of Chemistry and Biochemistry, University of California Los Angeles, Los Angeles, California, United States of America; 2 Department of Energy-Institute for Genomics and Proteomics, University of California Los Angeles, Los Angeles, California, United States of America; 3 Department of Chemical and Environmental Engineering, University of Arizona, Tuscon, Arizona, United States of America; 4 Chemistry Department, Brookhaven National Laboratory, Upton, New York, United States of America; 5 Department of Bioinspired Science, Ewha Womans University, Seoul, Korea; Instituto de Tecnologica Química e Biológica, UNL, Portugal

## Abstract

Two yeast manganese superoxide dismutases (MnSOD), one from *Saccharomyces cerevisiae* mitochondria (*Sc*MnSOD) and the other from *Candida albicans* cytosol (*Ca*MnSODc), have most biochemical and biophysical properties in common, yet *Sc*MnSOD is a tetramer and *Ca*MnSODc is a dimer or “loose tetramer” in solution. Although *Ca*MnSODc was found to crystallize as a tetramer, there is no indication from the solution properties that the functionality of *Ca*MnSODc *in vivo* depends upon the formation of the tetrameric structure. To elucidate further the functional significance of MnSOD quaternary structure, wild-type and mutant forms of *Sc*MnSOD (K182R, A183P mutant) and *Ca*MnSODc (K184R, L185P mutant) with the substitutions at dimer interfaces were analyzed with respect to their oligomeric states and resistance to pH, heat, and denaturant. Dimeric *Ca*MnSODc was found to be significantly more subject to thermal or denaturant-induced unfolding than tetrameric *Sc*MnSOD. The residue substitutions at dimer interfaces caused dimeric *Ca*MnSODc but not tetrameric *Sc*MnSOD to dissociate into monomers. We conclude that the tetrameric assembly strongly reinforces the dimer interface, which is critical for MnSOD activity.

## Introduction

Manganese-containing superoxide dismutase (SOD) enzymes catalyze the disproportionation of superoxide (O_2_^−^) into dioxygen and hydrogen peroxide (H_2_O_2_) and exist as either homodimers or homotetramers with a monomer size of ∼23,000 Da. Each MnSOD subunit is composed of two domains, a predominantly α-helical N-terminal domain and a mixed α/β C-terminal domain. The Mn ion is coordinated by four strictly conserved residues, two from the N-terminal domain and two from the C-terminal domain, as well as by one solvent molecule. The MnSOD functional unit is a dimer assembled in the fashion observed in prokaryotic MnSODs (*Thermus thermophilus* [1], *Escherichia coli* [2], [3], *Deinococcus radiodurans* [4]), while the MnSODs located in eukaryote mitochondria (human [5], *Saccharomyces cerevisiae* [6], *Caenorhabditis elegans* [7], *Aspergillus fumigatus* [8]) are homotetramers or dimers of dimers possessing dihedral symmetry (*D*_2_). The structure of the dimers within the tetrameric MnSODs is similar to those of the dimeric bacterial enzymes.

In previous studies, we compared two yeast MnSODs that share 58.3% sequence identity, one from *S. cerevisiae* mitochondria (*Sc*MnSOD), and an uncommon one from *Candida albicans* cytosol (*Ca*MnSODc) [9], and we demonstrated that *Sc*MnSOD and *Ca*MnSODc both exhibit faster catalysis than MnSODs from human or bacteria [9], [10], likely through a novel mechanism involving six-coordinate Mn^3+^ species [11]. The two yeast enzymes are also similar in terms of spectroscopy and redox chemistry [9]. To our surprise, *Ca*MnSODc was found to exist as a dimer in solution but as a tetramer when it was crystallized, whereas *Sc*MnSOD was found to be a tetramer under all conditions [9]. Because *Sc*MnSOD and *Ca*MnSODc have most biochemical and biophysical properties in common and their dimer interfaces share >90% similarity, they together provide an opportunity to probe the significance of the differing quaternary structures of MnSODs.

**Figure 1:**
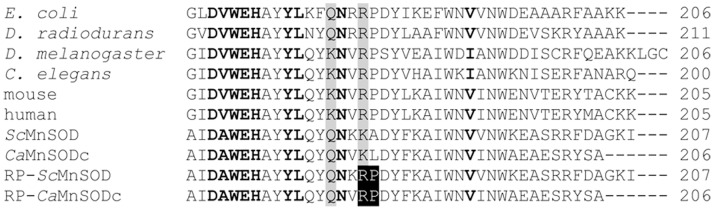
Alignment of MnSOD C-terminal Sequence. Conserved residues and unconserved residues at dimer interface are highlighted in bold and shadowed in gray, respectively. The RP-mutations in *Sc*MnSOD and *Ca*MnSODc are highlighted in black.

The dimer interface plays a crucial role in maintaining MnSOD activity. Most MnSODs have a conserved arginine close to the strictly conserved DXWEHXXYL motif, while in yeast MnSODs it is a lysine (Figure 1, Lys182 in *Sc*MnSOD and Lys184 in *Ca*MnSODc). Replacement of this lysine by arginine was reported to cause loss of stability but not of catalytic activity in *Sc*MnSOD [12]. This study, however, only measured the MnSOD activities at low levels of O_2_^−^.

Surprising findings that wild-type (WT) yeast MnSODs at high levels of O_2_^−^ are much faster enzymes than human and bacterial MnSODs have recently been reported [9], [10]. They gave impetus to our exploration of whether this discrepancy in catalytic behavior between yeast and human MnSOD is related to the different dimer interface structures. The side chain of the lysine in yeast MnSODs has a different conformation compared to that of the arginine in MnSODs from other organisms. This difference derives from the residue next to the lysine, which is an alanine or leucine in *Sc*MnSOD and *Ca*MnSODc, respectively, and a proline in all other MnSODs. To make yeast MnSODs resemble human MnSOD more closely and to investigate whether modifications of dimer interfaces has the same effects on tetrameric and dimeric MnSOD, we engineered the two yeast MnSODs by mutating the lysine to arginine and changing the residue next to the lysine to proline (K182R, A183P *Sc*MnSOD and K184R, L185P *Ca*MnSODc) (Figure 1). We call the mutant proteins RP-mutant MnSOD. We report here that, although the dimerization of the functional dimers to form a tetrameric assembly is not necessarily required for an eukaryotic MnSOD to function properly under physiological conditions, it preserves the dimeric functional unit and may protect MnSOD from deactivation and unfolding under harsh environments.

## Results

### The Mutations Create Holes at the Dimer Interface of RP-mutant *Sc*MnSOD and RP-mutant *Ca*MnSODc

**Figure 2:**
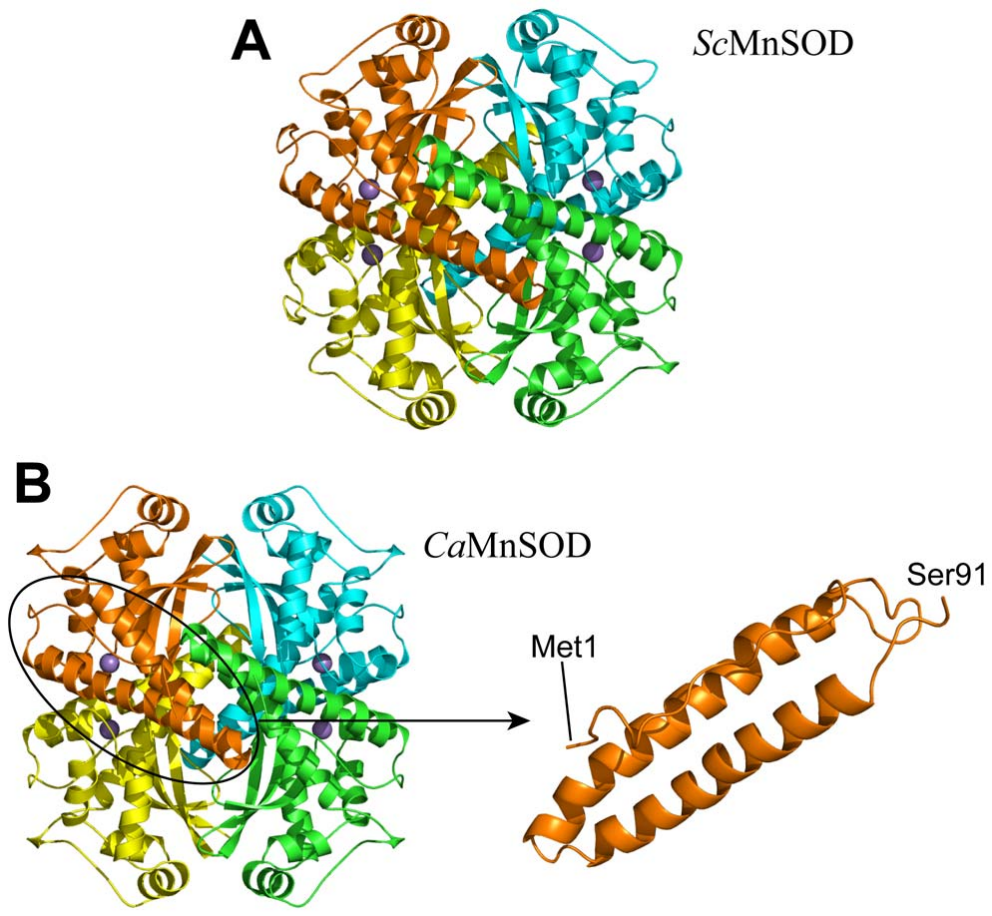
The tetramer interfaces are highly disordered, when *Ca*MnSODc is in the tetramer form. The ribbon diagram of *Sc*MnSOD (PDB code: 3LSU) is shown in Panel A. The four subunits are colored in: A, yellow; B, orange; C, green; D, cyan. The ribbon diagram of tetrameric *Ca*MnSODc (PDB code: 3QVN) and the N-terminal helical region (residues 1–91) of a *Ca*MnSODc monomer are shown in Panel B. The four subunits are colored in: A, yellow; B, orange; C, green; D, cyan. Manganese ions are indicated as purple spheres.

WT *Sc*MnSOD and WT *Ca*MnSODc and their RP-mutant proteins were overexpressed and purified from *S. cerevisiae*. Seeking clues as to why *Sc*MnSOD was a tetramer and *Ca*MnSODc a dimer or “loose tetramer” in solution, despite sharing a high sequence identity, we determined their crystal structures [9]. The crystal structure further confirmed that *Sc*MnSOD was a homotetramer (Figure 2A). By contrast, *Ca*MnSODc appeared as a homotetramer (Figure 2B) in crystal structures.

As in other tetrameric MnSODs, the N-terminus of each subunit of both yeast MnSODs folds into a hairpin structure holding two long α-helices (Figure 2A, 2B). The N-terminal helical hairpin in each of the yeast MnSODs is much longer than those in dimeric MnSODs from bacteria (Figure S1-B), and, in *Sc*MnSOD, it is longer than is found in any previously characterized tetrameric MnSOD (Figure S1-A). The buried surface areas at the tetramer interfaces are larger in *Sc*MnSOD and tetrameric *Ca*MnSODc (1417 and 1254 Å, respectively) than those (790–1000 Å^2^) in other MnSOD tetramers (human, *A. fumigates*, and *C. elegans*) (Table S2).

**Figure 3:**
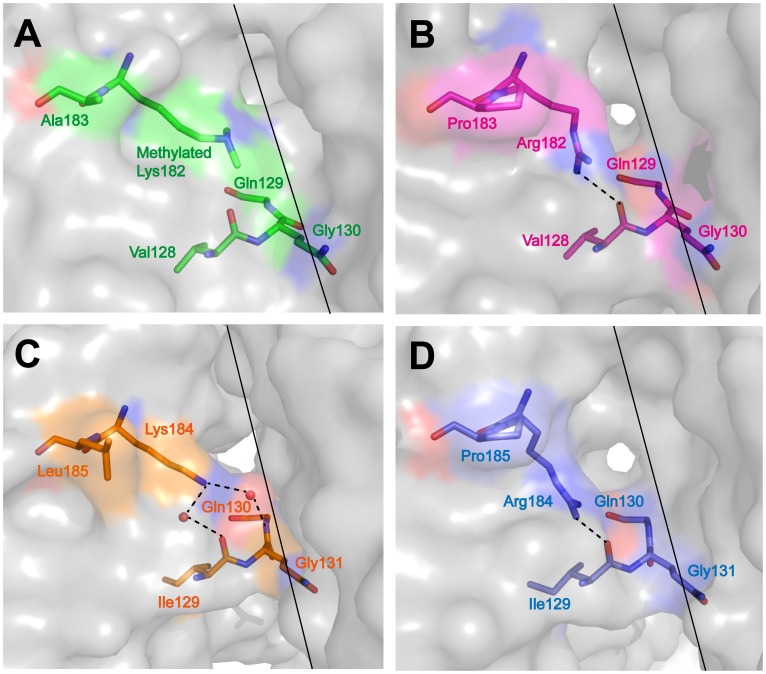
Comparison of the dimer interface surface structure of K182R, A183P *Sc*MnSOD and K184R, L185P *Ca*MnSODc to the WT proteins. The proteins are colored as: (A) WT *Sc*MnSOD, green; (B) K182R, A183P *Sc*MnSOD, red; (C) WT *Ca*MnSODc, orange; (D) K184R, L185P *Ca*MnSODc, blue. The dimer interfaces and hydrogen bonds are indicated as solid and dashed lines, respectively.

To investigate structural changes caused by the substitutions of Lys182 (Lys184) and Ala183 (Leu185) by arginine and proline, respectively, we solved the structures of the two RP-mutant proteins (Table 1). The tetrameric assemblies of WT and RP-mutant yeast MnSODs closely resemble each other (Figure S2). Superimpositions of all backbone atoms of the mutant subunit onto those of the WT subunit give root-mean-square deviations (RMSD) of 0.15 Å and 0.34 Å for *Sc*MnSOD and *Ca*MnSODc, respectively. The side chain of Arg182 (Arg184) of the mutants adopts a conformation different from that of Lys182 (Lys184) of the WT proteins (Figure 3). In two out of four chains in RP-mutant *Sc*MnSOD, the chi2 and chi3 angles of the arginine shift by 5 and 127°, respectively (Figure 3A, 3B). In the other two chains, the chi1 angle of the arginine shifts by 132° (data not shown). These changes move Arg182 (Arg184) away from the dimer interface and thus open up a hole at the dimer interface of both RP-mutant *Sc*MnSOD and RP-mutant *Ca*MnSODc (Figure 3). The mutations also modify the hydrogen-bonding interactions surrounding residue 182 (184). Two hydrogen bonds, NZ(Lys184)···O(solv)···O(Ile129) and NZ(Lys184)···O(solv)···N(Gly131), are observed in WT *Ca*MnSODc (Figure 3C), while in RP-mutant *Ca*MnSODc the arginine is hydrogen bonded to Ile129 (Figure 3D).

**Table 1:** X-ray Data Collection and Refinement Statistics^a^.

	K182R, A183P *Sc*MnSOD	K184R, L185P *Ca*MnSODc
PDB code	4F6E	4GUN
X-Ray source	Rigaku FRE+	Rigaku FRE+
Detector	Rigaku HTC	Rigaku HTC
Wavelength (Å)	1.5418	1.5418
Resolution range (Å)	37.65–1.60	53.02–1.94
R_sym_ (%)^b^	3.5 (17.0)	9.1 (29.5)
Reflections observed	400908	1206223
Unique reflections	105023	314901
Redundancy	3.8 (3.4)	3.8 (2.8)
I/σ	22.69 (6.79)	9.94 (3.21)
Completeness (%)	90.2 (70.8)	91.3 (64.4)
Space group	P1	P1
Unit cell a, b, c (Å)	65.54 66.25 66.62	129.33 73.80 134.29
Unit cell α, β, γ (°)	112.58 103.63 110.27	90.00 109.30 90.00
R_work_ (%)^c^	16.4 (19.2)	23.8 (23.5)
R_free_ (%)^c^	19.5 (23.3)	26.6 (27.4)
Wilson B value (Å^2^)	13.87	14.04
Protein molecules in asymmetric unit	4	16
Number of protein atoms	6665	25483
Number of non-protein atoms	573	1195
RMSD bond Lengths (Å)	0.006	0.007
RMSD bond Angles (°)	1.037	1.074
Average B-factor for protein atoms (Å^2^)	16.47	14.85
Average B-factor for non-protein atoms (Å^2^)	25.19	14.28
Ramachandran angles		
Most favored (%)	91.8	92.2
Additionally allowed (%)	7.1	6.7
Generously allowed (%)	1.1	0.7
Disallowed (%)	0.0	0.4

a Highest resolution shell shown in parenthesis.

b R_sym_ = Σ*_hkl_* |*I_hkl_*−<*I_hkl_*>|/Σ*_hkl_I_hkl_*.

c R_factor_ = Σ||*F_obs_*|−|*F_calc_*||/Σ|*F_obs_*|. R_work_ refers to the R_factor_ for the data utilized in the refinement and R_free_ refers to the R_factor_ for 5% of the reflections randomly chosen that were excluded from the refinement.

### RP-Mutant Yeast MnSODs have WT Dismutase Activities

As-isolated proteins partially loaded with Mn (Table S1) were used in biophysical studies from this point, because the apoproteins of both WT and RP-mutant yeast MnSODs are unstable and titration of metal ions into as-isolated proteins has not been successful. As-isolated WT and RP-mutant *Sc*MnSOD contain 0.70 and 0.71 Mn per monomer, respectively. As-isolated WT and RP-mutant *Ca*MnSODc contain 0.59 and 0.43 Mn per monomer, respectively. SOD activities are reported on a per metal ion basis.

As noted earlier, the two yeast MnSODs were engineered through residue substitutions to imitate human MnSOD. To explore whether these residue substitutions affected the catalytic activity of yeast MnSODs, WT and RP-mutant yeast enzymes were pulse irradiated with various concentrations of O_2_^−^ and their dismutation efficiencies were compared. At neutral pH and room temperature, the disappearances of O_2_^−^ in the presence of WT and mutant proteins follow similar kinetics. Even when [O_2_^−^] was high relative to enzyme concentration ([O_2_^−^]:[MnSOD] = 41), the decay curves for O_2_^−^ disappearance catalyzed by WT or RP-mutant yeast MnSODs were superimposable (Figure S3), suggesting that the mutant proteins resemble the WT enzymes in displaying low degrees of product inhibition [9], [10]. Although our size exclusion chromatography experiments indicate that RP-mutant *Ca*MnSODc could partially dissociate into monomers at 1.0 µM (see below), pulse radiolysis experiments suggest that it was more likely to stay predominantly as a dimer or “loose tetramer” at that concentration. Its rate constant determined at 1 µM (9.1×10^8^ M^−1^s^−1^) was already near diffusion controlled and only slightly increased (9.9×10^8^ M^−1^s^−^1) when the enzyme concentration was increased to 2.2 µM.

**Figure 4:**
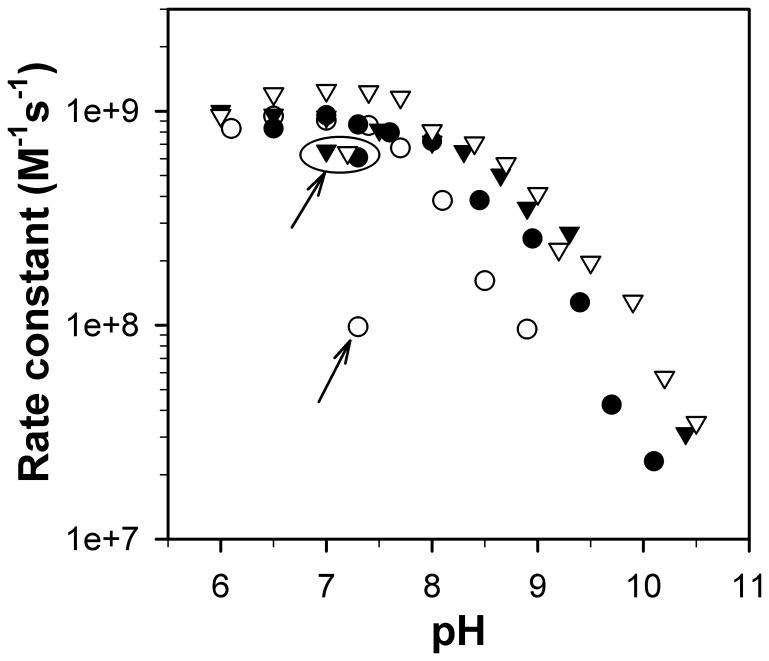
RP-mutant *Ca*MnSODc is more subject to inactivation by pH than the wild type. Rate constants as a function of pH were determined by fitting the disappearances of low doses of O_2_^−^ ([O_2_^−^]:[MnSOD] from 1–3) to first-order processes. The enzymes were WT *Sc*MnSOD (solid triangle), K182R, A183P *Sc*MnSOD (hollow triangle), WT *Ca*MnSODc (solid circle) and K184R, L185P *Ca*MnSODc (hollow circle). The data points circled and/or indicated with an arrow were measured after the pH was adjusted from 9–9.5 to neutral. The sample solutions contained 1 µM (in Mn) MnSOD in 10 mM potassium phosphate (pH 7), 10 mM sodium formate and 10 µM EDTA.

MnSOD catalytic activity (reactions 1 and 2) was measured when [O_2_^−^]:MnSOD ratio ranged from 1–3 to exclude any effect of product inhibition. We previously showed that inactivation occurs at a significantly lower pH in yeast MnSODs than in human MnSOD, with p*Ks* (the pH at which the SOD activity drops by 50%) of 8.5 and 10.5, respectively [9]. Here, although both yeast MnSODs were engineered to imitate human MnSOD, neither of the mutant proteins gained stability at higher pH compared to the WT enzymes (Figure 4). The profile of RP-mutant *Sc*MnSOD activity as a function of pH closely resembles that of WT *Sc*MnSOD (Figure 4). The same mutations on *Ca*MnSODc, however, resulted in an enzyme even more sensitive to pH, with the p*K* decreasing from ∼8.5 in the wild type to ∼8 in the mutant protein (Figure 4). Loss of activity at high pH was found to be reversible in WT yeast enzymes as well as in RP-mutant *Sc*MnSOD, with a restoration of ∼50% of their original activity (Figure 4). By contrast, when the pH of the sample solution was adjusted from basic (≥9) to neutral, no restoration of activity was observed for RP-mutant *Ca*MnSODc (Figure 4).

### RP-mutant *Ca*MnSODc is Inactivated by Heat, While RP-mutant *Sc*MnSOD is not

**Figure 5:**
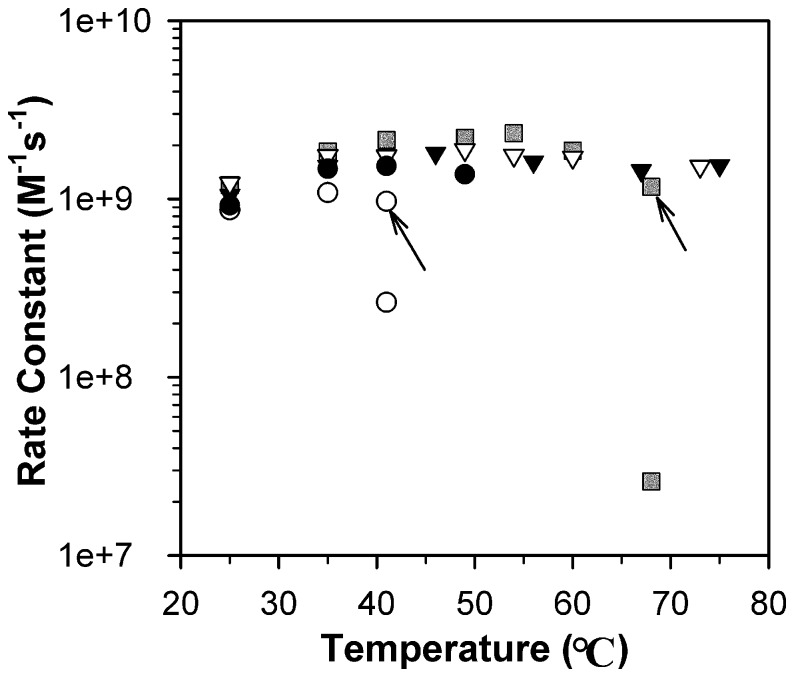
RP-mutant *Ca*MnSODc is inactivated by heat like *Ec*MnSOD. Rate constants as a function of pH were determined by fitting the disappearances of low doses of O_2_^−^ ([O_2_^−^]:[MnSOD] from 1–3) to first-order processes. The enzymes were *Ec*MnSOD (grey rectangle), WT *Sc*MnSOD (solid triangle), K182R, A183P *Sc*MnSOD (hollow triangle), WT *Ca*MnSODc (solid circle) and K184R, L185P *Ca*MnSODc (hollow circle). The data points indicated with an arrow were obtained before the sample solution reached the desired temperature. All other data points were obtained after the sample solution was equilibrated to the desired temperature. The sample solutions contained 1 µM (in Mn) MnSOD in 10 mM potassium phosphate (pH 7), 10 mM sodium formate and 10 µM EDTA.

Yeast MnSODs showed full activity until the protein unfolding temperature. *Sc*MnSOD was fully active up to 75°C, the highest temperature allowed in pulse radiolysis measurements. *Ca*MnSODc, with a much lower thermostability than *Sc*MnSOD (see below), was fully active as long as the enzyme stayed folded in solution. Protein aggregation was noticeable when the sample became more opaque to light at 260 nm. Aggregation of as-isolated *Ca*MnSODc occurred at 50°C (see below), and the enzyme stayed fully active up to 49°C (Figure 5).

By contrast, MnSOD from *E. coli* (*Ec*MnSOD) has been known for being inactivated by heat. Indeed, the reactivity of *Ec*MnSOD decreased by ∼100 fold after the sample solution was allowed to equilibrate to 68°C (Figure 5, see method). Because there was no evidence of sample opacity at 260 nm, the dramatic inactivation by heat in *Ec*MnSOD appears to result from some mechanism other than aggregation of the enzyme.

The substitutions of Lys182 (Lys184) and Ala 183 (Leu185) at dimer interfaces were found to affect the activities of *Sc*MnSOD and *Ca*MnSODc at elevated temperatures differently. Similar to WT *Sc*MnSOD, RP-mutant *Sc*MnSOD retained full activity up to 73°C (Figure 5). RP-mutant *Ca*MnSODc, however, differed from the wild type but closely resembled the bacterial MnSODs with respect to its inactivation by heat. When RP-mutant *Ca*MnSODc was heated at 41°C, a loss of ∼70% activity was observed (Figure 5). RP-mutant *Ca*MnSODc started aggregating at ∼46°C (see below) but remained soluble at 41°C.

### RP-mutant *Ca*MnSODc is Susceptible to Dimer Dissociation

**Figure 6:**
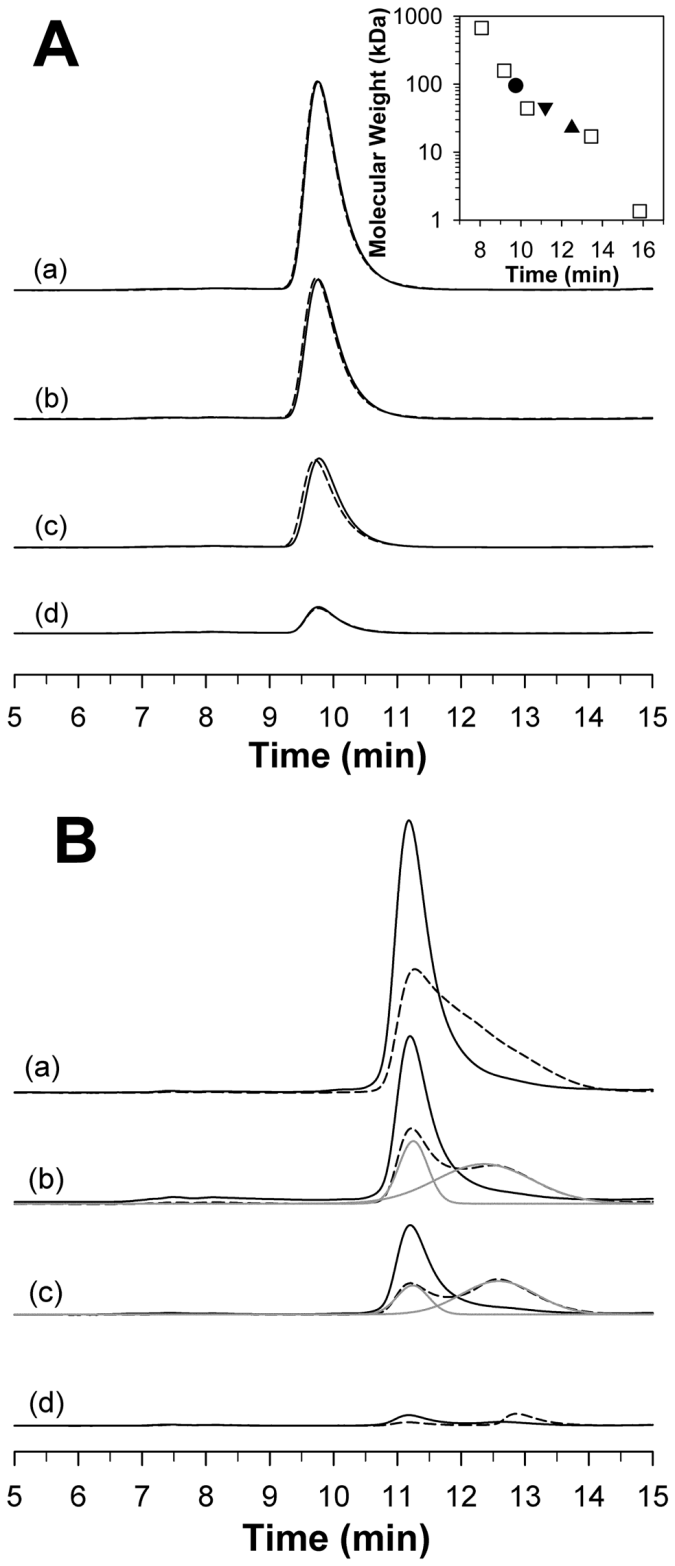
RP-mutant *Ca*MnSODc is susceptible to dimer dissociation. HPLC-SEC profiles of WT (solid line) and K182R, A183P (dashed line) *Sc*MnSOD are shown in Panel A. Inset: The plot of the molecular weight of the five standards (square), *Sc*MnSOD tetramer (circle) and *Ca*MnSODc dimer (triangle down) and monomer (triangle up) versus their retention time. The column was calibrated using five standards: 1) bovine thyroglobulin (670 kDa), 2) bovine γ-globulin (158 kDa), 3) ovalbumin (44 kDa), 4) horse myoglobin (17 kDa), and 5) Vitamin B12 (1.35 kDa). HPLC-SEC profiles of WT (solid line) and K184R, L185P (dashed line) *Ca*MnSODc are shown in Panel B. Deconvoluted peaks are shown in grey lines. The protein concentration relative to monomer was 1 µM (a), 750 nM (b), 500 nM (c) and 200 nM (d). The elution buffer contained 10 mM potassium phosphate (pH 6.7).

The oligomeric states of the as-isolated proteins were investigated by HPLC-SEC. When the protein concentration with respect to monomer was varied from 10 µM to 200 nM, WT and RP-mutant *Sc*MnSOD both eluted solely as tetramers (Figure 6A), and WT *Ca*MnSODc eluted solely as dimers (Figure 6B). The different oligomeric states of WT *Sc*MnSOD and WT *Ca*MnSODc are unlikely to result from differences in metallation states (Table S1), since WT *Sc*MnSOD and WT *Ca*MnSODc, when both metallated with ∼0.6 Mn per monomer, elute as tetramers and dimers, respectively [9].

The elution profiles of RP-mutant *Ca*MnSODc revealed two peaks corresponding to dimeric and monomeric forms when the protein concentration with respect to monomer was below 1 µM (Figure 6B). At 200 nM RP-mutant *Ca*MnSODc, only the monomeric form was observed (Figure 6B). Based on the dimer-monomer equilibrium (Materials and Methods), *K*_d_ of as-isolated RP-mutant *Ca*MnSODc was determined to be 2.0±0.1 µM, with details of the calculation shown in Table S3. However, we cannot exclude the possibility that difference in metallation states (Table S1) might affect the dimer-monomer equilibrium in RP-mutant *Ca*MnSODc.

### WT and RP-mutant *Ca*MnSODc are More Susceptible to Denaturant-Induced Protein Unfolding than WT and RP-mutant *Sc*MnSOD

**Figure 7:**
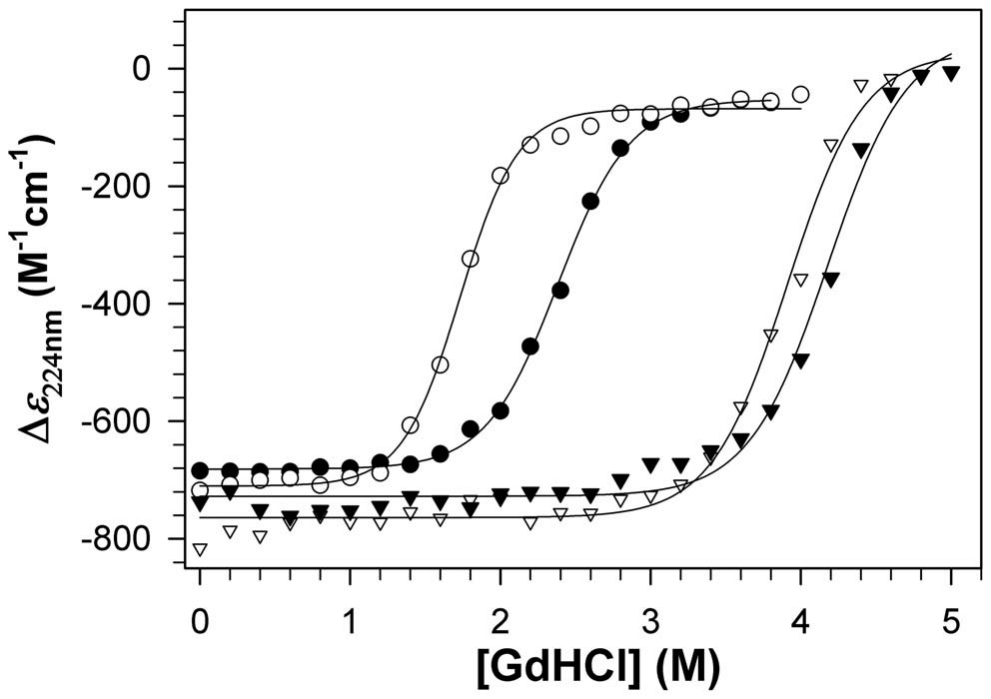
RP-mutant *Ca*MnSODc is more subject to GdHCl-induced unfolding than the wild type. The molar CD at 224 nm was used to monitor changes in α-helical structure content as a function of [GdHCl]. The enzymes were WT *Sc*MnSOD (solid triangle), K182R, A183P *Sc*MnSOD (hollow triangle), WT *Ca*MnSODc (solid circle) and K184R, L185P *Ca*MnSODc (hollow circle). The sample solutions contained 0.2 mg/mL (monomer concentration) MnSOD in 25 mM potassium phosphate (pH 7.4).

In order to understand the impact of the quaternary structure and the dimer interface on MnSOD stability, we used CD spectroscopy to monitor the denaturant-induced unfolding transitions of WT and RP-mutant yeast MnSODs (Figure S4). The molar CD at 224 nm was used to monitor changes in α-helical structure content as a function of [GdHCl] (Figure 7). Under these experimental conditions, *Sc*MnSOD is a tetramer while *Ca*MnSODc is a dimer or “loose tetramer”. The sharp decrease in helical structure content occurred at ∼3.4 M GdHCl in *Sc*MnSOD and ∼1.6 M GdHCl in *Ca*MnSODc (Figure 7). The unfolding of RP-mutant *Ca*MnSODc occurred at a lower concentration (∼1.2 M) of GdHCl than that of WT *Ca*MnSODc (Figure 7). By contrast, the unfolding profiles of WT and RP-mutant *Sc*MnSOD are comparable to each other (Figure 7).

### WT and RP-mutant *Ca*MnSODc are Significantly Less Thermally Stable than WT and RP-mutant *Sc*MnSOD

**Figure 8:**
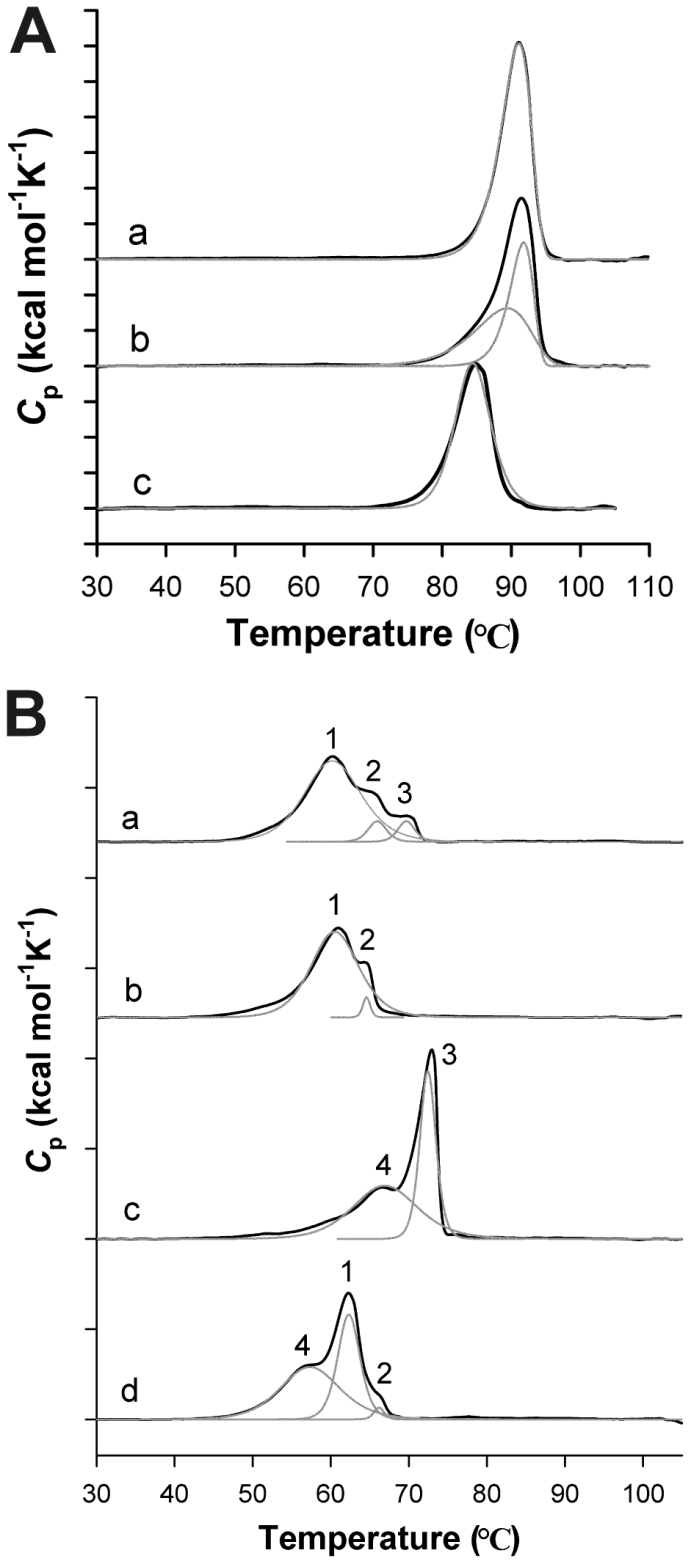
Thermostability of WT and RP-mutant *Sc*MnSOD and *Ca*MnSODc. The *S. cerevisiae* enzymes in (A) are: (a) as-isolated *Sc*MnSOD, (b) oxidized *Sc*MnSOD and (c) as-isolated K182R, A183P *Sc*MnSOD. The *C. albicans* enzymes in (B) are: (a) as-isolated *Ca*MnSODc; (b) reduced *Ca*MnSODc; (c) oxidized *Ca*MnSODc and (d) as-isolated K184R, L185P *Ca*MnSODc. Unfolding transitions are shown in black lines. The components (gray) were deconvoluted using a two-state irreversible model for WT *Sc*MnSOD and a non-two-state reversible model for RP-mutant *Sc*MnSOD, and WT and RP-mutant *Ca*MnSODc. Reduced or oxidized enzymes were prepared by adding sodium hyposulfite or potassium permanganate to the sample solution prior to the DSC scan.

To investigate the impact of the quaternary structure and the residue substitutions at dimer interfaces on MnSOD thermostability, we monitored the unfolding transitions of WT and RP-mutant yeast MnSODs by DSC (Materials and Methods). Although the heat treatment of all WT and RP-mutant yeast MnSODs led to irreversible aggregation of the proteins, the DSC profiles were fitted using either a two-state irreversible model or a non-two-state reversible model, depending on which model yielded a better fitting. WT and mutant proteins are both partially loaded with Mn (Table S1). WT and RP-mutant *Sc*MnSOD are loaded with 0.70 and 0.71 Mn per subunit, respectively. WT and RP-mutant *Ca*MnSODc are loaded with 0.59 and 0.43 Mn per subunit, respectively. All four rest predominantly in the reduced (2+) state. The DSC profile of as-isolated *Sc*MnSOD showed a single transition at 91°C, corresponding to one irreversible process (Figure 8A–a). This suggests cooperativity in the aggregation of apo and metallated subunits in *Sc*MnSOD.

By contrast, three endotherms with much lower *T*_m_s were observed upon heat treatment of as-isolated *Ca*MnSODc, assigned as Therm 1, 2, and 3 (Figure 8B–a, Table 2). In order to assign peaks with confidence, we measured the DSC profile for fully reduced *Ca*MnSODc (Figures 8B–b). Therm 2 likely resulted from a small portion of Fe-containing SOD (Table S1) as it is retained in both fully reduced and as-isolated *Ca*MnSODc. Because the activity of MnSOD is Mn-specific [13], [14], we assume that this small portion of Fe-substituted protein does not contribute to the activity of *Ca*MnSODc. Since Therm 3 was missing in the thermal stability profile of reduced *Ca*MnSODc (Figure 8B–b), it correlated with the aggregation of the oxidized Mn^3+^-*Ca*MnSODc in the as-isolated enzyme. Therm 1 corresponded to the cooperative melting of apo- and Mn^2+^-containing *Ca*MnSODc.

**Table 2:** Thermodynamic Parameters for Unfolding of Yeast MnSODs.

	Component Number	*T*_m_ (°C)	Δ*H* a (kcal per mole of monomer)
As-isolated *Sc*MnSOD^b^		91.0	705
Oxidized *Sc*MnSOD^b^		91.5	659
As-isolated *Ca*MnSODc^c^	1	60.3	431
As-isolated *Ca*MnSODc^c^	2	66.0	36
As-isolated *Ca*MnSODc^c^	3	69.7	33
Reduced *Ca*MnSODc^c^	1	60.9	353
Reduced *Ca*MnSODc^c^	2	64.5	13
Oxidized *Ca*MnSODc^c^	4	66.5	325
Oxidized *Ca*MnSODc^c^	3	72.9	254
As-isolated K182R, A183P *Sc*MnSOD^c^		84.4	563
As-isolated K184R, L185P *Ca*MnSODc^c^	1	57.4	288
As-isolated K184R, L185P *Ca*MnSODc^c^	2	62.3	216
As-isolated K184R, L185P *Ca*MnSODc^c^	3	66.2	12

a Δ*H* are given per mole of tetramer or dimer.

b The peaks of the DSC profile were deconvoluted using an irreversible two-state model.

c The peaks of the DSC profile were deconvoluted using a reversible non-two-state model.

The stability profiles of yeast MnSODs are affected by the metal oxidation states. Chemically oxidized (3+) *Ca*MnSODc gave a wide therm (4) prior to the emergence of Therm 3 (Figure 8B–c). To explore the origin of Therm 4, oxidized *Ca*MnSODc was heated at 65°C for 20 min, and the metal content of the resulting supernatant was determined by ICP-MS. Because the proteins in the supernatant were nearly fully metallated with Mn, Therm 4 was likely associated with the aggregation of apo subunits in oxidized *Ca*MnSODc. The separation of apoprotein endotherm from holoprotein endotherm was more subtle in oxidized *Sc*MnSOD, although its main transition occurred at a lower temperature (∼75°C) than the as-isolated protein (∼80°C) and could well be deconvoluted to two irreversible processes (Figure 8A–b).

Like the WT enzyme, as-isolated RP-mutant *Sc*MnSOD gave a single endotherm with a *T*_m_ lowered to 84°C (Figure 8A–c). By contrast, the DSC profile of as-isolated RP-mutant *Ca*MnSODc deviated more dramatically from that of WT *Ca*MnSODc. Because nearly fully metallated protein remained in the supernatant upon heat treatment of RP-mutant *Ca*MnSODc at 57°C, the endotherm at 57°C likely resulted from the aggregation of apo-RP-*Ca*MnSODc, which, unlike in WT *Ca*MnSODc, was separated from that of the aggregation of holo-RP-*Ca*MnSODc (Figure 8B–d). The mutant protein maintained the two therms at 62 and 66°C, corresponding to the aggregation of Mn^2+^-containing and Fe-substituted *Ca*MnSODc, respectively (Figure 8B–d, Table 2).

## Discussion

### Tetramerization is not Required for *Ca*MnSODc to Function under Physiological Conditions

The two yeast MnSODs, one from *S. cerevisiae* mitochondria and the other from *C. albicans* cytosol, share 58.3% sequence identity. *Sc*MnSOD is always a homotetramer like human MnSOD [5], [9]. To our surprise, *Ca*MnSODc is a dimer or “loose tetramer” in solution (Figure 6B), and it is a tetramer when crystallized (Figure 2B). The biophysical characterization showing that *Ca*MnSODc is significantly less stable than *Sc*MnSOD (Figure 8) confirms that in solution *Sc*MnSOD and *Ca*MnSODc are tetrameric and dimeric, respectively. However, from their crystal structures (Figure 2) we cannot explain the discrepancy in their quaternary structures. Tetrameric MnSODs are in equilibrium between dimers and tetramers, and this kind of equilibrium is usually dependent on factors such as ionic strength, temperature, pH, and concentration of denaturant [15], [16]. Our results suggest that the tetramer-dimer equilibrium lies toward dimers in *Ca*MnSODc, while it lies toward tetramers in other tetrameric MnSODs (MnSOD from *S. cerevisiae*, human, *C elegans* or *A. fumigatus*).

Because the helical hairpins contain two of the active site ligands, it was previously hypothesized that the tetramer interface played a role in stabilizing the helical hairpins and that only tetrameric enzymes would be active and stable [5]. Indeed, the I58T or L60F (tetramer interface residue) variants of human MnSOD have a much shorter half-life than the wild type at increased temperatures [17], [18]. Numerous other tetrameric proteins and enzymes with dihedral symmetry, such as malic enzyme, chaperone SecB, and RUNX1/ETO fusion protein, display impaired function when dissociating into dimers or monomers [19]–[21]. By contrast, *Ca*MnSODc was in the dimeric form in our kinetics studies, yet its catalytic properties resemble those of tetrameric *Sc*MnSOD [9]. Our studies suggest that the propensity for tetramerization found for eukaryotic but not for prokaryotic MnSODs is not related to optimization of SOD activity. A similar phenomenon has also been reported for aristolochene synthase from *Aspergillus terreus*, which functions as a dimer in solution, but is capable of tetramerization at high enzyme concentrations [22].

It is doubtful that tetrameric *Ca*MnSODc significantly surpasses dimeric *Ca*MnSODc in reactivity, because the latter is already near diffusion-controlled [9]. Dimeric *Ca*MnSODc could be the active form that functions *in vivo*. However, since it is difficult to compare the composition of *C. albicans* cytosol to the *in vitro* conditions, we are unable to elucidate the *in vivo* quaternary structure of *Ca*MnSODc.

### The Tetramer Structure Reinforces the Dimer Interface

Although *Ca*MnSODc is indistinguishable from *Sc*MnSOD in terms of enzyme kinetics, spectroscopy, and redox properties [9], dimeric *Ca*MnSODc is considerably less stable than tetrameric *Sc*MnSOD. Compared to tetrameric *Sc*MnSOD, the unfolding of dimeric *Ca*MnSODc occurs at a much lower level of denaturant (GdHCl) (Figure 7), and the *T*_m_ of the main components of dimeric *Ca*MnSODc is lower by 30°C. These features suggest an important role of the tetramerization domain in enhancing the MnSOD enzyme’s resistance against unfolding in harsh environments.

Several studies suggest a significant role for the dimer interface in both catalysis and stability of MnSOD. Replacement of Glu170 in *Ec*MnSOD, which spans the dimer interface and forms the double glutamate bridge, results in dissociation of the dimer, complete loss of catalytic activity, and a change in metal specificity [23]. Substitutions at Glu162 in human MnSOD, the counterpart of Glu170 in *Ec*MnSOD, reduce the catalytic activity to 5–25% of that of the WT enzyme [24]. The Y166F mutant human MnSOD shows a significant decrease in catalytic activity and a major unfolding transition at a lower *T*_m_ [25]. Replacement of Phe66 at the dimer interfaces of human MnSOD reduces the degree of product inhibition in the human enzyme and makes it resemble *Ec*MnSOD [26], [27].

Here, two residues (Lys182 and Ala183 in *Sc*MnSOD, Lys184 and Leu185 in *Ca*MnSODc) were substituted at the dimer interface of the two yeast MnSODs. We show here that these two RP-mutant proteins resemble their WT enzymes in terms of SOD activity at room temperature and neutral pH (Figure S3) so that the roles of these two residues appear to be related to protein stability, not enzymatic function.

Even though the former is a tetramer and the latter is a dimer in solution, *Sc*MnSOD and *Ca*MnSODc, have >90% sequence similarity at the dimer interface and share most biochemical and biophysical characteristics. Based on our findings, we conclude that the dimer interface of RP-mutant *Ca*MnSODc is considerably destabilized. The dimer dissociation constant, *K*_d_, which is too low to measure in WT *Ca*MnSODc, significantly increases to 2.0±0.1 µM in the mutant protein. RP-mutant *Ca*MnSODc also becomes more sensitive to high pH than the wild type, and this inactivation by increased pH becomes completely irreversible (Figure 4). In contrast to WT *Ca*MnSODc, the RP-mutant protein exhibits loss of activity at high temperatures (Figure 5), and it is more subject to unfolding by GdHCl (Figure 7). These observations suggest that the mutations at the dimer interface cause *Ca*MnSODc dimers to fall apart into monomers.

The destabilization of the dimer interface in RP-mutant *Ca*MnSODc is also suggested by the DSC protein stability data. The oxidized form of *Ca*MnSODc has a higher thermal stability than the reduced form (Figure 8B). The dependence of protein stability on the oxidation state was also reported in *Ec*MnSOD, which has a greater affinity for Mn^3+^ [28]. Based on the DSC data in Figure 8B and Table 1, the molar energy required for aggregation of each enzyme species in WT *Ca*MnSODc scales as Mn^3+^-containing *Ca*MnSODc>Fe-substituted *Ca*MnSODc>Mn^2+^-containing *Ca*MnSODc ∼ apo subunits. Removal of the metal ions from WT or RP-mutant *Ca*MnSODc causes precipitation of the proteins, suggesting that metal-free *Ca*MnSODc is not stable. Therefore, since as-isolated WT and RP-mutant *Ca*MnSODc contain ∼0.59 and ∼0.43 Mn per monomer (Table S1), respectively, each *Ca*MnSODc dimer likely contains one metal ion. As the Mn,E-*Ca*MnSODc dimer is heated, the apo subunits melt at 57–60°C (Table 2). The remaining manganese-bound subunits then self-associate to create the Mn,Mn-*Ca*MnSODc dimer, the aggregation of which occurs at 65–73°C, depending on the oxidation state of the manganese (Table 2). This phenomenon has been reported for another type of SOD, copper-zinc SOD loaded with one or two zinc ions [29]. In RP*-*mutant *Ca*MnSODc, the order of the molar energies changes to Fe-substituted *Ca*MnSODc>Mn^2+^-containing *Ca*MnSODc>apo subunits (Figure 8B–d, Table 2). The modifications of dimer interfaces lower the energy threshold for dimer dissociation and thus make aggregation of apo subunits occur more readily in RP-mutant *Ca*MnSODc.

The mutations do not result in considerable perturbation in the subunit structure of the two yeast enzymes (Figure S2). Examination of the mutated residues, however, suggests that Arg182 (Arg184) in the mutants moves away from the dimer interface, which creates a hole at the dimer interface and would likely cause the destabilization of the dimer interface in both the mutants (Figure 3). Nevertheless, the same residue substitutions at dimer interfaces have much milder effects on tetrameric *Sc*MnSOD, although WT *Sc*MnSOD and WT *Ca*MnSODc are similar in many ways [9]. Unlike RP-mutant *Ca*MnSODc, which dissociates into monomers, RP-mutant *Sc*MnSOD closely resembles the wild type in oligomeric state, and resistance to pH, heat, and denaturant (Figure 4–7, S3). The only sign of destabilization is the slightly lower thermal stability displayed by the mutant protein relative to the wild type (Figure 8A).

Assembly of proteins into oligomer structures has been proposed to confer several potential advantages, including greater folding efficiency, high stability, specific morphological functions, amenability to allosteric regulation, and greater error control in synthesis [15], [16], [30]. Tetramer interface has been reported to contribute to thermal and pH-dependent stability of numerous dihedral tetrameric enzymes, including malate dehydrogenase and serine hydroxymethyltransferase [31]–[33]. Specifically in tetrameric MnSODs, each side of the protein is encircled by one of the two 4-helix bundles at opposite ends of the dimer, which acts as a clamp, holding the dimers in place. In addition to the contribution to protein stability, our results here suggest that the dimer interface, which is critical for MnSOD activity, is strongly reinforced through tetramer formation.

In conclusion, the tetramer-dimer equilibrium of *Ca*MnSODc lies toward dimers in solution, and its activity under physiological conditions does not rely upon tetramerization. Thus the significance of the tetramer structure may lie in the stabilization of protein assembly against harsh environments (heat and denaturant). More importantly, our results suggest that tetrameric assembly of the functional dimers strongly reinforces the functional dimer interface. As the functional dimer interface is critical for MnSOD activity, its reinforcement through tetramerization could be one of the reasons that MnSOD is tetrameric in higher organisms.

## Materials and Methods

### Samples

*E. coli* MnSOD (*Ec*MnSOD) was purchased from a commercial source (Sigma-Aldrich). The protein was resuspended in 25 mM potassium phosphate (pH 7.4), washed with 1 mM EDTA in the same phosphate buffer for several times, and then purified through a G200 size exclusion column.

### Construction of Plasmid for Expression of RP-mutant *Sc*MnSOD and RP-mutant *Ca*MnSODc

Site-directed mutagenesis [34] was carried out on an overexpression vector (YEp352-*Sc*MnSOD) containing the *URA*3 selectable marker and a 2-kb genomic *Bam*HI fragment containing the gene for *Sc*MnSOD. The primers 5′-CAGTACCAAAACAAG**AGACCC**GACTACTTCAAAGC-3′ and 5′-GCTTTGAAGTAGTC**GGGTCT**CTTGTTTTGGTACTG-3′ were used to create the cDNA for K182R, A183P *Sc*MnSOD.

The pVT102U-*Ca*MnSODc (with *URA3* and *AMP* marker) vector containing the complete coding sequence of *Ca*MnSODc was generously given by Prof. Bourbonnais [35]. The primers 5′-CAAAATGTC**AGGCCT**GATTATTTCAAAGCAATTTGGAACGTG-3′ and 5′-GAAATAATC**AGGCCT**GACATTTTGATATTGCAAGTAGTACGC-3′ were used to create the cDNA for K184R, L185P *Ca*MnSODc.

The PCR products were transformed into *E. coli DH5α* strain and screened by ampicillin selection. The purified vectors were transformed into *S. cerevisiae sod2Δ* strain (EG110).

### Expression and Purification of WT and RP-mutant *Sc*MnSOD and *Ca*MnSODc

Yeast cells carrying YEp352-*Sc*MnSOD (WT or mutant) were grown in YPEG media (1% yeast extract, 2% peptone, 3% glycerol, 2% ethanol, pH 4) supplemented with 0.5 mM Mn(II) sulfate at 30°C to O.D. >20. Yeast cells carrying pVT102U-*Ca*MnSODc (WT or mutant) were grown in YPD (1% yeast extract, 2% peptone, 2% dextrose, pH 4) media supplemented with 0.5 mM Mn(II) sulfate at 30°C to O.D. >10. Cells were harvested by centrifugation at 12,000×g for 10 min. Isolation of WT and RP-mutant *Sc*MnSOD and *Ca*MnSODc was performed as previously described [9].

### Size Exclusion Chromatography

The mass weight of native proteins was determined by a HPLC (Agilent 1200 series) fitted with a size exclusion column (Tosoh Bioscience, TSK gel G2000SW) at a flow rate of 0.25–0.5 mL/min. The column was calibrated using five standards: bovine thyroglobulin (670 kDa), bovine γ-globulin (158 kDa), ovalbumin (44 kDa), horse myoglobin (17 kDa), and vitamin B12 (1.35 kDa).

HPLC-SEC measurements were used to determine the oligomeric state of proteins. The equilibrium between dimers and monomers is shown as 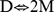 where D represents dimer and M represents monomer. The dimer dissociation constant (*K*_d_) is calculated as
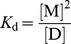
(1)where [M] and [D] were calculated from area integrals of elution peaks (UV signal at 210 nm). The peak fitting was carried out in Origin 8.1 (OriginLab Corp.). The column buffer contained 10 mM potassium phosphate (pH 6.7). The protein concentration with respect to monomer was varied from 10 µM to 200 nM.

### Crystallization of WT and RP-mutant *Sc*MnSOD and *Ca*MnSODc

The crystallization of both WT *Sc*MnSOD and WT *Ca*MnSODc, giving tetrameric structures, was described previously [9]. Reductive methylation of K182R, A183P *Sc*MnSOD was carried out as described previously [36], in order to improve the diffraction of protein crystals. All free amino groups of the lysine residues and the N-terminus of each subunit of the two proteins were methylated as confirmed by mass spectrometry (Figure S5).

Methylated K182R, A183P *Sc*MnSOD, containing 0.71 Mn per monomer (Table S1), was crystallized by hanging-drop vapor diffusion at 4°C against a well solution of 0.1 M tri-ammonium citrate (pH 7) in 20% (w/v) polyethylene glycol 3,350 with a protein concentration of 7 mg/mL. Unmodified K184R, L185P *Ca*MnSODc, containing 0.43 Mn per monomer (Table S1), was crystallized by hanging-drop vapor diffusion at 4°C against a solution of 2 M ammonium sulfate with a protein concentration of 7 mg/mL. The protein crystals were cryo-protected in mother liquor solution containing 30% glycerol and flash frozen in liquid nitrogen prior to data collection. These two structures were deposited to PDB bank (see below).

### Crystallography: Data Collection and Refinement

The crystallography information for WT *Sc*MnSOD and WT *Ca*MnSODc was reported previously [9]. The data of RP-mutant *Sc*MnSOD and RP-mutant *Ca*MnSODc were collected at 100 K at the UCLA X-ray diffraction facility, using a Rigaku FRE+ generator and a Rigaku HTC detector. The data was processed using XDS [37]. RP-mutant *Sc*MnSOD and RP-mutant *Ca*MnSODc were phased by molecular replacement using WT *Sc*MnSOD (PDB code: 3LSU) and WT *Ca*MnSODc (PDB code: 3QVN), respectively. All the molecular replacements were done using PHASER [38]. The models were built using COOT [39]. All model refinements were performed using REFMAC [40], PHENIX [41] and BUSTER [42].

Coordinates and structure factors have been deposited in the PDB database with accession numbers 4F6E for the K182R, A183P *Sc*MnSOD structure and, 4GUN for the K184R, L185P *Ca*MnSODc structure. Analysis of interface contacts was performed using the PISA server (PDBePISA Protein Interfaces, Surfaces and Assemblies [43].

### Pulse Radiolysis

Pulse radiolysis experiments were carried out using the 2 MeV Van de Graaff accelerator at Brookhaven National Laboratory. Upon irradiation of water with a pulse of energetic electrons, hydrated electrons (e_aq_^−^), hydroxyl radicals (^•^OH) and, in lesser yield, hydrogen atoms (H^•^) are the primary radicals produced. Superoxide radical is then generated in air-saturated aqueous solution containing sodium formate through the following reactions: ^•^OH+HCO_2_^−^ → H_2_O+CO_2_^•−^, O_2_+ CO_2_^•−^ → O_2_^•−^+CO_2_, e_aq_^−^+O_2_ → O_2_^•−^, H+O_2_ → HO_2_^•^.

The experiments to measure catalytic rates involved following the decay of various concentrations of O_2_^−^ at 260 nm using 1∶1 to 1∶50 ratios of [MnSOD]:[O_2_^−^]. First-order rate constants were calculated by fitting the data using the kinetics program in PRWIN [44].

The thermal deactivation measurements were performed by heating the pulse radiolysis cell holder to the desired temperature. The sample cell containing a protein solution was then inserted into the holder and allowed to equilibrate for 4 minutes prior to irradiation of the sample solution.

All pulse radiolysis samples were prepared in 10 mM potassium phosphate, 10 mM sodium formate and 10 µM EDTA at 25°C. All MnSOD concentrations were taken as the ICP-measured concentration of manganese in the sample. The pH of the buffer was adjusted using ultrapure (Baker Ultrex) sodium hydroxide and sulfuric acid as needed.

### Differential Scanning Calorimetry (DSC)

DSC scans were performed using a Nano II differential scanning calorimeter (Calorimetry Sciences Corp.). All buffers were degassed under vacuum and highly concentrated protein samples (∼24 mg/mL) were diluted with degassed buffers to a concentration of 2 mg/mL (600 *µ*L) right before the DSC run. All samples were run at a rate of 1°C/min under 4 atm of pressure. A buffer (25 mM potassium phosphate, pH 7.4) base line was run prior to the analysis of protein samples with the same heating/cooling rates. Baseline subtraction was performed, and some of the peaks were fitted to non-two-state reversible transitions using Origin 8.0 (OriginLab Corp.).

The other peaks were fitted to two-state irreversible transitions, obeying pseudo-first-order kinetics [45]. The temperature dependence of the kinetic constant *k* obeys the Arrhenius equation,
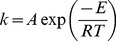
(2)where *A* is the preexponential factor and *E* is the activation energy of transition. The apparent excess heat capacity *C*_p_(*T*) at a temperature *T* is given by Sánchez-Ruiz’s methods as
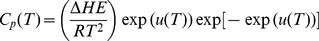
(3)where 
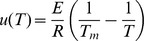
, Tm is the temperature at which Cp reaches its maximum value. DSC data were fitted to equation (3) using SigmaPlot 11.0 (Systat Software).

### Miscellaneous Methods

Metal contents of the purified proteins were determined by inductive coupled plasma mass spectrometry (ICP-MS, Agilent 7500 series). UV-visible absorption spectra were collected on a Shimadzu UV-2501 PC spectrophotometer.

Circular dichroism (CD) measurements were performed on a JASCO J-715 spectropolarimeter at room temperature. The GdHCl concentration was varied from 0 to 5 M while the protein concentration was held constant at 0.2 mg/mL. Protein samples were mixed with GdHCl stock solutions and incubated at room temperature for 15 min prior to the scan.

Mass weight of the protein subunit was determined by electrospray ionization mass spectrometry (ESI-MS) either via a triple quadrupole instrument (API III, Applied Biosystems) or via a hybrid linear ion-trap mass spectrometer (LTQ, Thermo Electron). Mass spectra were processed and analyzed using MacSpec 3.3, Hypermass and BioMultiview 1.3.1 software for data obtained from API III, and ProMass for Xcalibur 2.8 for data obtained from LTQ.

## Supporting Information

Figure S1 Superimposition of the subunit of *Sc*MnSOD (green) over that of human (pink) and *E. coli* (orange) MnSOD. The two subunits are colored in: A, green; B, cyan.
(TIF)

Figure S2 Comparison of crystal structures between WT yeast MnSODs and their RP-mutant proteins. (A and B). Superimposition of the tetramer of K182R, A183P *Sc*MnSOD (pink) and K184R, L185P *Ca*MnSODc (blue) to that of WT *Sc*MnSOD (green) and *Ca*MnSODc (orange), respectively. (C and D). Superimposition of the monomer of K182R, A183P *Sc*MnSOD (pink) and K184R, L185P *Ca*MnSODc (blue) to that of WT *Sc*MnSOD (green) and *Ca*MnSODc (orange), respectively. Manganese atoms are shown in purple spheres.
(TIF)

Figure S3 Decay of 43 µM O_2_^−^ catalyzed by (A) WT *Sc*MnSOD (solid, a), K182R, A183P *Sc*MnSOD (dotted, b), (B) WT *Ca*MnSODc (solid, a) and K184R, L185P *Ca*MnSODc (dotted, b). The sample for pulse radiolysis contains 10 mM phosphate (pH 7), 10 mM sodium formate and 10 µM EDTA. The O_2_^−^ concentration in these figures was calculated from the absorbance at 260 nm and is slightly different from the O_2_^−^ dose given by the pulse radiolysis instrument.
(TIF)

Figure S4 CD spectra of WT *Ca*MnSODc (A), K184R, L185P *Ca*MnSODc (B), WT *Sc*MnSOD (C) and K182R, A183P *Sc*MnSOD (D) at increased concentrations of GdHCl. The solutions contained 25 mM potassium phosphate (pH 7.4). The measurements were carried out at room temperature.
(TIF)

Figure S5 Electrospray-ionization mass spectra of methylated RP-mutant *Sc*MnSOD (left) and its reconstructed mass distribution profile (right). Ordinate units of intensity are arbitrary and the abscissa units of average molecular mass are in Daltons. The theoretical mass weight of methylated K182R, A183P *Sc*MnSOD is 23,589 Da.
(EPS)

Table S1 Metal contents of WT and RP-mutant *Sc*MnSOD and *Ca*MnSODc.
(DOC)

Table S2 Interactions of Subunits at Dimer and Tetramer Interfaces in MnSODs from Different Organisms.
(DOC)

Table S3 Calculation of *K*_d_ for K184R, L185P *Ca*MnSODc.
(DOC)
