# Supplementary figures and images for: Tetramerization Reinforces the Dimer Interface of MnSOD
Source: PLoS One. 2013 May 7;8(5):e62446. doi: 10.1371/journal.pone.0062446 (PMC3646814; doi:10.1371/journal.pone.0062446)

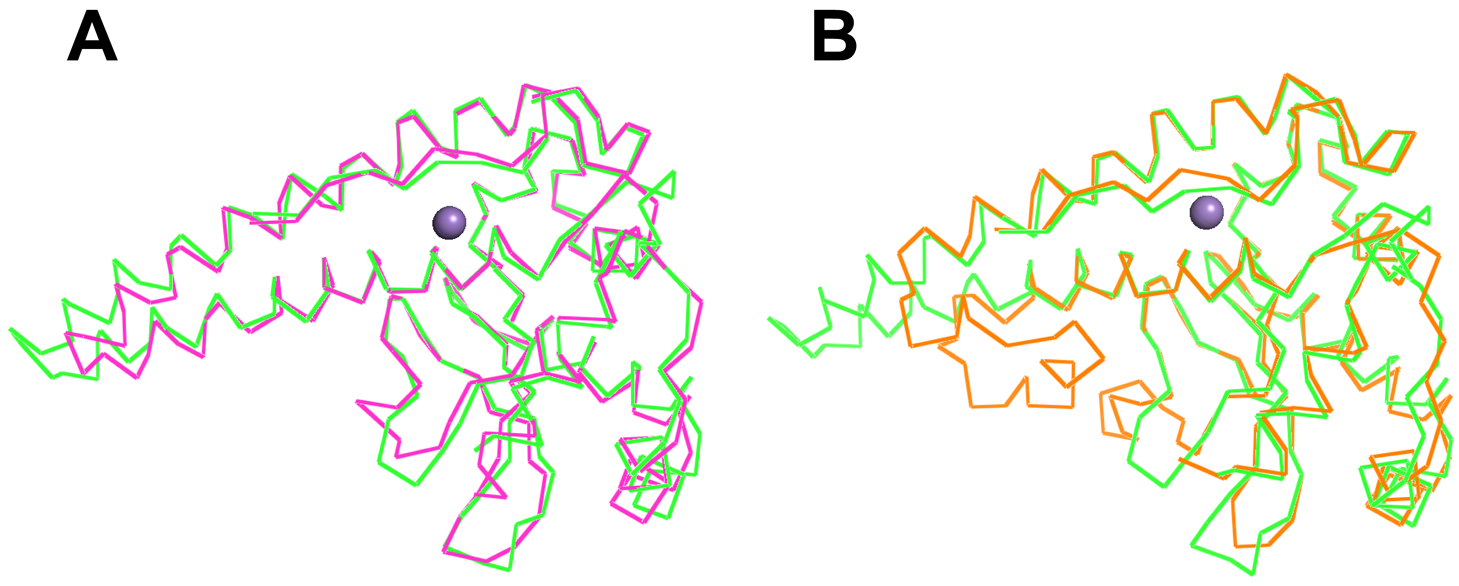

Supplement: Figure S1 — Superimposition of the subunit of ScMnSOD (green) over that of human (pink) and E. coli (orange) MnSOD. The two subunits are colored in: A, green; B, cyan. (TIF) [file pone.0062446.s001.tif]

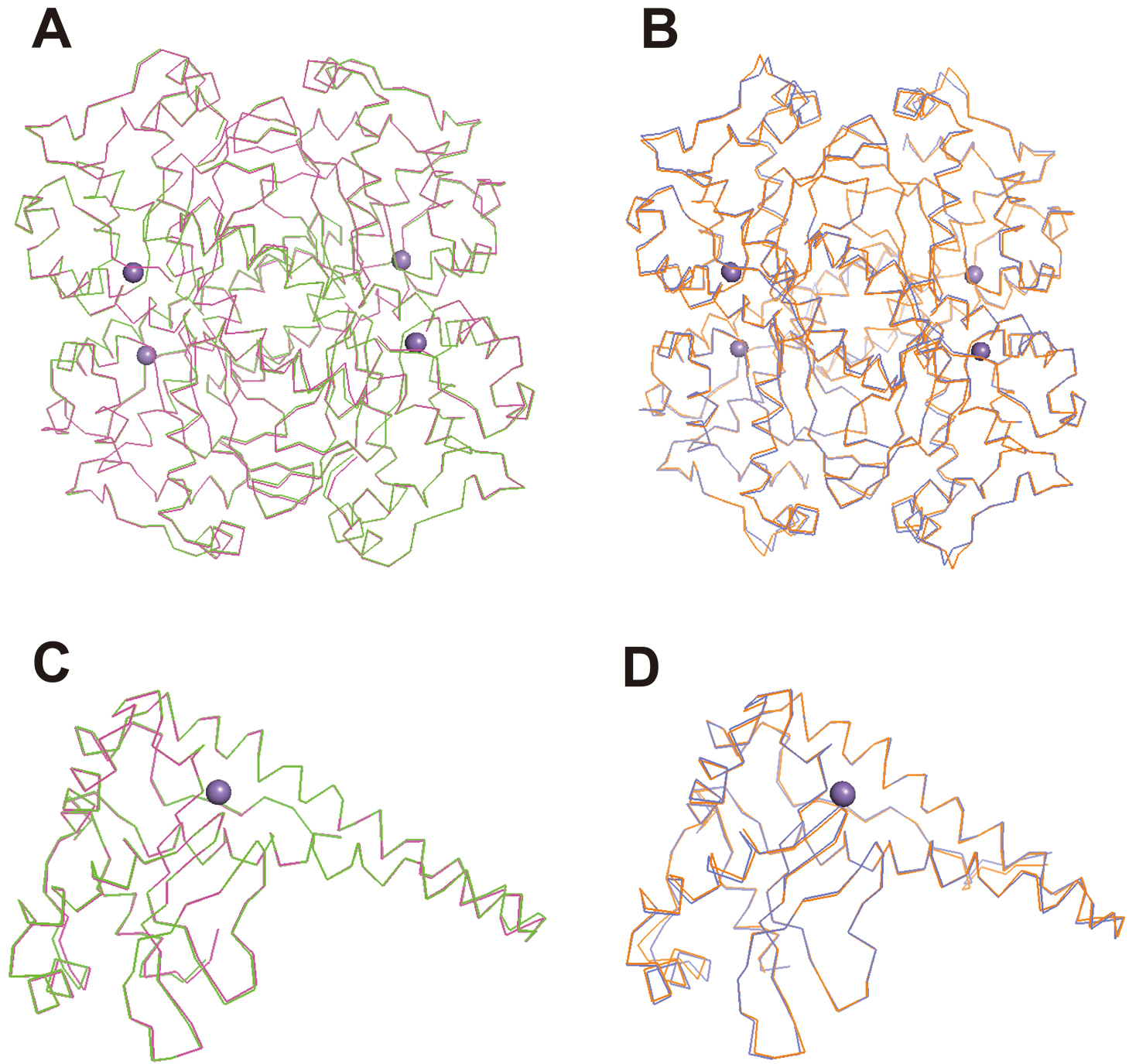

Supplement: Figure S2 — Comparison of crystal structures between WT yeast MnSODs and their RP-mutant proteins. (A and B). Superimposition of the tetramer of K182R, A183P ScMnSOD (pink) and K184R, L185P CaMnSODc (blue) to that of WT ScMnSOD (green) and CaMnSODc (orange), respectively. (C and D). Superimposition of the monomer of K182R, A183P ScMnSOD (pink) and K184R, L185P CaMnSODc (blue) to that of WT ScMnSOD (green) and CaMnSODc (orange), respectively. Manganese atoms are shown in purple spheres. (TIF) [file pone.0062446.s002.tif]

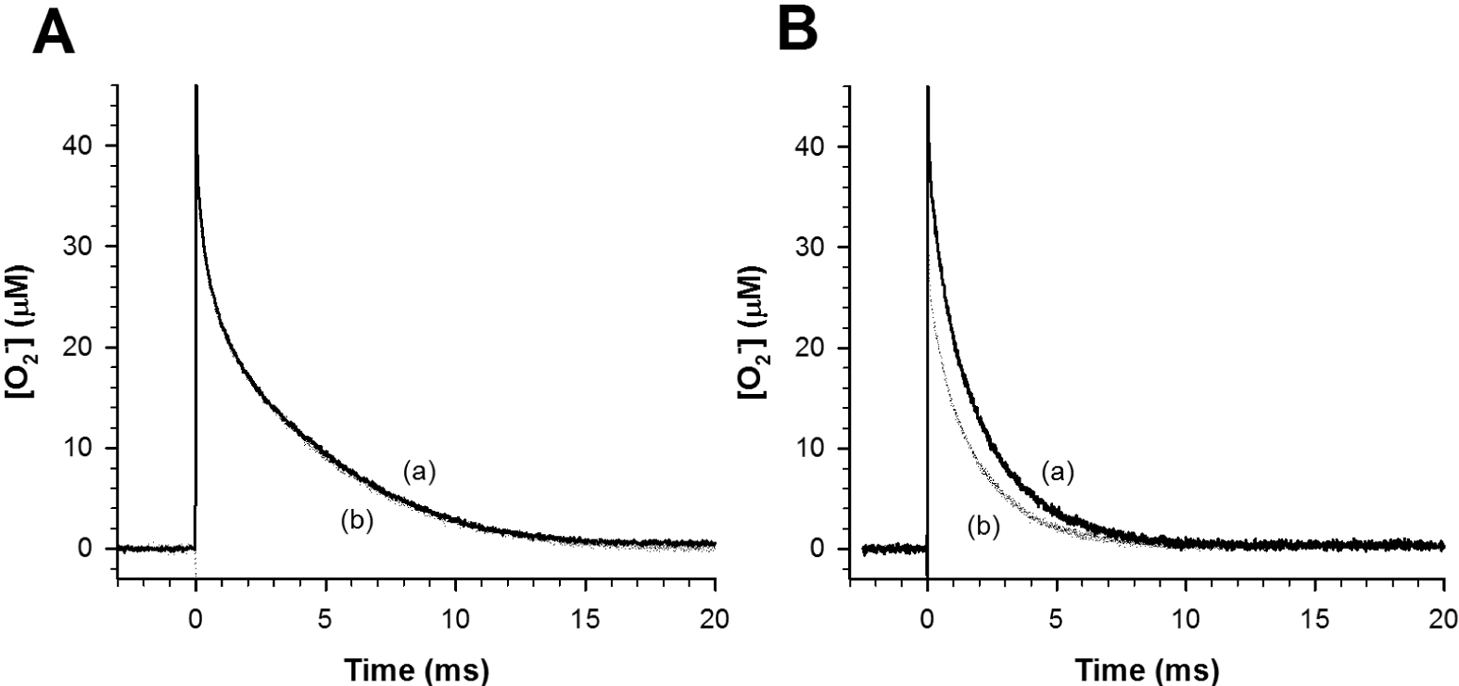

Supplement: Figure S3 — Decay of 43 µM O2 − catalyzed by (A) WT ScMnSOD (solid, a), K182R, A183P ScMnSOD (dotted, b), (B) WT CaMnSODc (solid, a) and K184R, L185P CaMnSODc (dotted, b). The sample for pulse radiolysis contains 10 mM phosphate (pH 7), 10 mM sodium formate and 10 µM EDTA. The O2 − concentration in these figures was calculated from the absorbance at 260 nm and is slightly different from the O2 − dose given by the pulse radiolysis instrument. (TIF) [file pone.0062446.s003.tif]

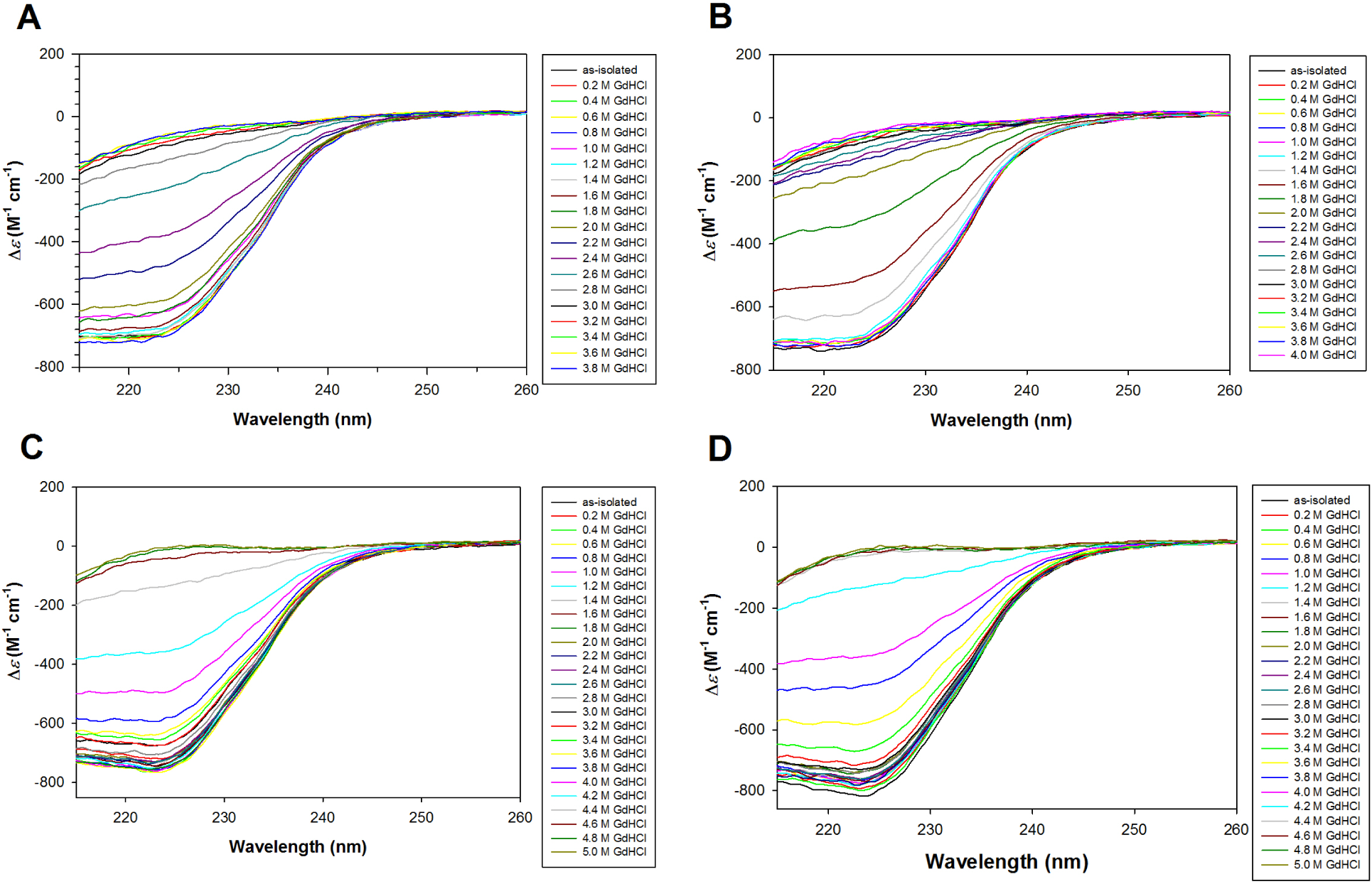

Supplement: Figure S4 — CD spectra of WT CaMnSODc (A), K184R, L185P CaMnSODc (B), WT ScMnSOD (C) and K182R, A183P ScMnSOD (D) at increased concentrations of GdHCl. The solutions contained 25 mM potassium phosphate (pH 7.4). The measurements were carried out at room temperature. (TIF) [file pone.0062446.s004.tif]
