# Supplementary material for: Tetramerization Reinforces the Dimer Interface of MnSOD
Source: PLoS One. 2013 May 7;8(5):e62446. doi: 10.1371/journal.pone.0062446 (PMC3646814; doi:10.1371/journal.pone.0062446)
Supplement: Table S1 — Metal contents of WT and RP-mutant ScMnSOD and CaMnSODc. (DOC) [file pone.0062446.s006.doc]

Table S1. Metal contents of WT and RP-mutant *Sc*MnSOD and *Ca*MnSODc

|  | Mn per monomer | Fe per monomer |
| --- | --- | --- |
| WT *Sc*MnSOD | 0.70 | 0.04 |
| K182R, A183P *Sc*MnSOD | 0.71 | 0.05 |
| WT *Ca*MnSODc | 0.59 | 0.06 |
| K184R, L185P *Ca*MnSODc | 0.43 | 0.12 |
