# Supplementary material for: Tetramerization Reinforces the Dimer Interface of MnSOD
Source: PLoS One. 2013 May 7;8(5):e62446. doi: 10.1371/journal.pone.0062446 (PMC3646814; doi:10.1371/journal.pone.0062446)
Supplement: Table S2 — Interactions of Subunits at Dimer and Tetramer Interfaces in MnSODs from Different Organisms. (DOC) [file pone.0062446.s007.doc]

Table S2. Interactions of Subunits at Dimer and Tetramer Interfaces in MnSODs from Different Organisms

|  | *Sc*MnSOD | *Ca*MnSODc  (tetramer) | human WT | *A. fumigatus* | *C. elegans* | *E. coli* WT | *T. Thermophilus* | *Drad* WT |
| --- | --- | --- | --- | --- | --- | --- | --- | --- |
| Mn•••Mn (A/B, dimer, Å) | 18.2 (0.04) | 18.3 | 18.3 | 18.1 (0.1) | 18.3 | 18.4 (0.01) | 18.4 | 18.3 |
| Mn•••Mn (A/C, tetramer, Å) | 39.7 (0.2) | 38.4 | 42.0 | 39.8 (0.1) | 41.6 |  |  |  |
| Mn•••Mn (A/D, tetramer, Å) | 38.7 (0.04) | 38.4 | 41.0 | 38.7 (0.2) | 40.3 |  |  |  |
| Dimer interface area (Å^2^) | 956 | 840 | 844 | 883 | 864 | 858 | 878 | 906 |
| Tetramer interface area (Å^2^) | 1417 | 1254 | 961 | 797 | 800 |  |  |  |
