# Supplementary material for: Tetramerization Reinforces the Dimer Interface of MnSOD
Source: PLoS One. 2013 May 7;8(5):e62446. doi: 10.1371/journal.pone.0062446 (PMC3646814; doi:10.1371/journal.pone.0062446)
Supplement: Table S3 — Calculation of K d for K184R, L185P CaMnSODc. (DOC) [file pone.0062446.s008.doc]

Table S3. Calculation of *K*_d_ for K184R, L185P *Ca*MnSODc

| Protein Concentration | 750 nM | | 500 nM | |
| --- | --- | --- | --- | --- |
|  | 1 | 2 | 1 | 2 |
| Area integral of dimer peak (mAU×s) | 1405 | 1519 | 777 | 755 |
| Area integral of monomer peak (mAU×s) | 2939 | 3216 | 2114 | 1932 |
| Concentration of dimer (nM) | 121.3 | 120.3 | 67.2 | 70.3 |
| Concentration of monomer (nM) | 507.5 | 509.4 | 365.6 | 359.5 |
| *K*_d_ | 2.1 μM | 2.2 μM | 2.0 μM | 1.8 μM |
|  |  |  |  |  |
| Average *K*_d_ | 2.0 ± 0.1 μM | | | |
